# ECFS-DEA: an ensemble classifier-based feature selection for differential expression analysis on expression profiles

**DOI:** 10.1186/s12859-020-3388-y

**Published:** 2020-02-05

**Authors:** Xudong Zhao, Qing Jiao, Hangyu Li, Yiming Wu, Hanxu Wang, Shan Huang, Guohua Wang

**Affiliations:** 1 College of Information and Computer Engineering, Northeast Forestry University, No.26 Hexing Road, Harbin, 150040 China; 2 Department of Neurology, The 2nd Affiliated Hospital of Harbin Medical University, No. 246 Xuefu Road, Harbin, 150086 China; 3 State Key Laboratory of Tree Genetics and Breeding, Northeast Forestry University, No.26 Hexing Road, Harbin, 150040 China

**Keywords:** Feature selection, Classification, Accumulation, Expression profiles, Differential expression analysis

## Abstract

**Background:**

Various methods for differential expression analysis have been widely used to identify features which best distinguish between different categories of samples. Multiple hypothesis testing may leave out explanatory features, each of which may be composed of individually insignificant variables. Multivariate hypothesis testing holds a non-mainstream position, considering the large computation overhead of large-scale matrix operation. Random forest provides a classification strategy for calculation of variable importance. However, it may be unsuitable for different distributions of samples.

**Results:**

Based on the thought of using an **e**nsemble **c**lassifier, we develop a **f**eature **s**election tool for **d**ifferential **e**xpression **a**nalysis on expression profiles (i.e., ECFS-DEA for short). Considering the differences in sample distribution, a graphical user interface is designed to allow the selection of different base classifiers. Inspired by random forest, a common measure which is applicable to any base classifier is proposed for calculation of variable importance. After an interactive selection of a feature on sorted individual variables, a projection heatmap is presented using k-means clustering. ROC curve is also provided, both of which can intuitively demonstrate the effectiveness of the selected feature.

**Conclusions:**

Feature selection through ensemble classifiers helps to select important variables and thus is applicable for different sample distributions. Experiments on simulation and realistic data demonstrate the effectiveness of ECFS-DEA for differential expression analysis on expression profiles. The software is available at http://bio-nefu.com/resource/ecfs-dea.

## Background

Differential expression analysis (DEA) is widely adopted to identify a feature for best characterizing the expression difference between groups of individuals (e.g., healthy ones and those affected with a disease) [1]. Multiple hypothesis testing, which evaluates more than one hypothesis simultaneously, plays an important role in DEA. Corresponding tools such as SAM [2], limma [3], multtest [4], etc. have been produced for detecting differentially expressed variables. As a matter of fact, multiple hypothesis testing may leave out an explanatory signature. A selected feature expressed differently may not be composed of individually significant variables [5]. Although multivariate hypothesis testing may choose a proper feature, it still holds a non-mainstream position [6], considering the need for a large computation overhead of large-scale matrix operation.

Unlike statistical hypothesis testing, classification-based feature selection concentrates on better classification results of a certain subspace in many aspects such as sequence analysis [7, 8], site identification [9–12], protein classification [13, 14], protein identification [15, 16], protein fold recognition [17–19], protease substrate prediction [20, 21] and protein backbone torsion angle prediction [22]. Thus, predictive variables [23–25] are selected according to classification results of a certain classifier. Random forest [26, 27] is a case in point. It utilizes decision trees as the base classifier, which may be unsuitable for different distributions of samples. We have developed JCD-DEA [28], which is a feature selection tool combining hypothesis testing with classification strategy. However, JCD-DEA employs a bottom-up feature enumeration strategy, which is time consuming.

In this paper, we develop a top-down classification-based feature selection tool, i.e. ECFS-DEA, for differential expression analysis. In addition to random forest (RF), one of the other three classifiers, i.e., Fisher’s linear discriminant analysis (LDA), k-nearest-neighbor (kNN) and support vector machine (SVM), can be interactively selected to be the base classifier in accordance with different sample distributions. Under the development environment of Python 3.5, ECFS-DEA applicable to various execution environments such as a personal computer, a workstation or a large-scale cluster in Windows, Linux or Mac, can be used to identify the feature which best distinguishes between different categories of samples on expression profiles such as RNA-seq data, microarrays, etc.

## Method

**Fig. 1:**
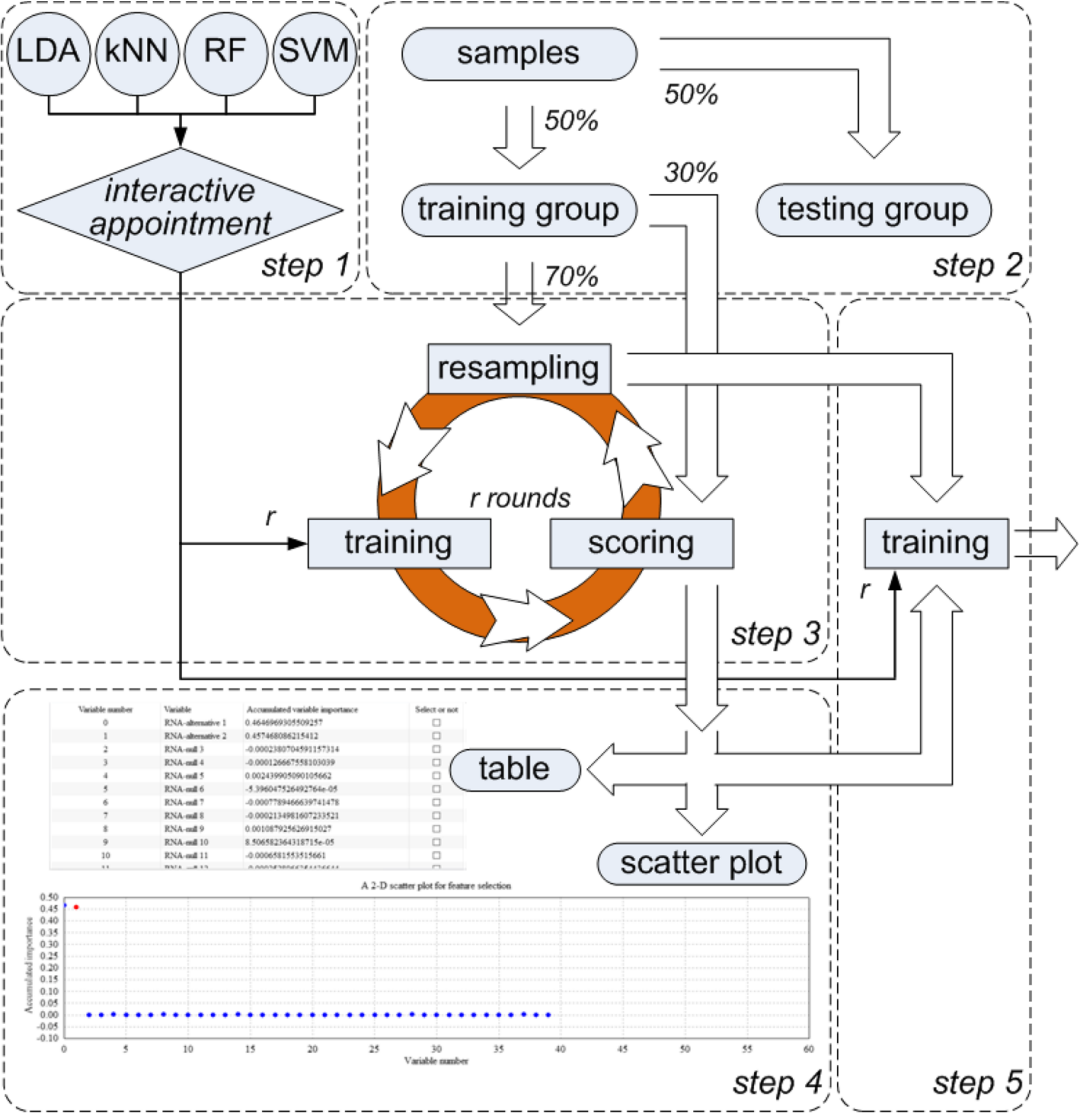
Schematic of feature selection part in ECFS-DEA

ECFS-DEA offers two main functions, i.e. feature selection and feature validation. Feature selection part contains five steps, as illustrated in Fig. 1. Firstly, the category of the base classifier is to be interactively appointed. RF, LDA, kNN and SVM are the alternative base classifier. The base classifier number *r* is also to be set. Meanwhile, the path of the input file, the data format and the execution environment are to be selected. Secondly, samples are randomly divided into training and testing groups in balance. Thirdly, a resampling procedure is constructed for the accumulation of variable importance. The resampling round is equivalent to the number of the base classifiers. In each round *j*, 70% of training samples are randomly selected in the entire feature space for training each classifier; while, the remaining 30% of training samples are the out-of-bag data for calculating the classification error rate *Err*_*j*_. As to each variable *i*, only one time permutation of its expression levels on the out-of-bag data is made, and the corresponding classification error rate is presented as $Err^{0}_{j}(i)$. After *r* rounds of resampling, the importance of variable *i* is achieved as $\sum _{j=1}^{n}\left (Err_{j}^{0}(i)-Err_{j}\right)/r$. Fourthly, a feature can be manually selected in a table with the individual variables sorted in descending order according to achieved variable importance or in a 2-D scatter plot with its horizontal and vertical coordinates corresponding to the variable indices and the accumulated importance, respectively. Fifthly, an ensemble classifier composed of *r* same base classifiers is to be trained using the expression levels of the training samples on the selected feature.

As to feature validation part, the testing samples are needed. Aiming at the expression levels of the testing set on the selected feature, a scatter plot in 1-D, 2-D or 3-D subspace can be illustrated. The corresponding ROC curve is also provided. Besides, a projection heatmap which displays discrete projection values (i.e., classification results) from the expression levels of the selected feature, is presented. Using the trained classifier, the classification results of the testing set on the selected feature are reordered based on k-means clustering. Accompanied with the expression levels and the labels, the reordered classification results are shown in the projection heatmap.

## Implementation

ECFS-DEA is written mainly in Python 3.5, distributed under GNU GPLv3. Considering the existence of repeating steps in ECFS-DEA, we make a two-step implementation: a client part in *Client.zip* for executing GUI, and a server part in *Server.zip* which is designed to run on the cluster server that using Portable Batch System(PBS) as scheduling program. The client part also contains codes for analyzing expression profiles, if ECFS-DEA can only run on a personal computer or a workstation.

**Fig. 2:**
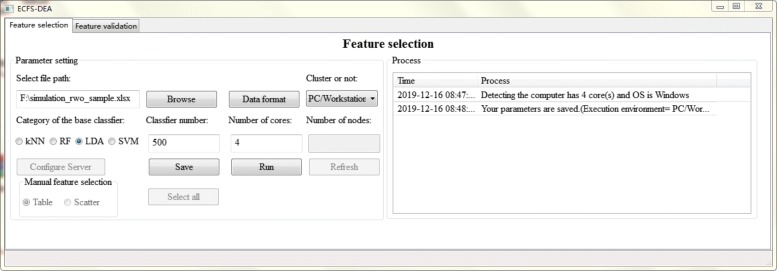
The parameter setting step of feature selection part in ECFS-DEA

**Fig. 3:**
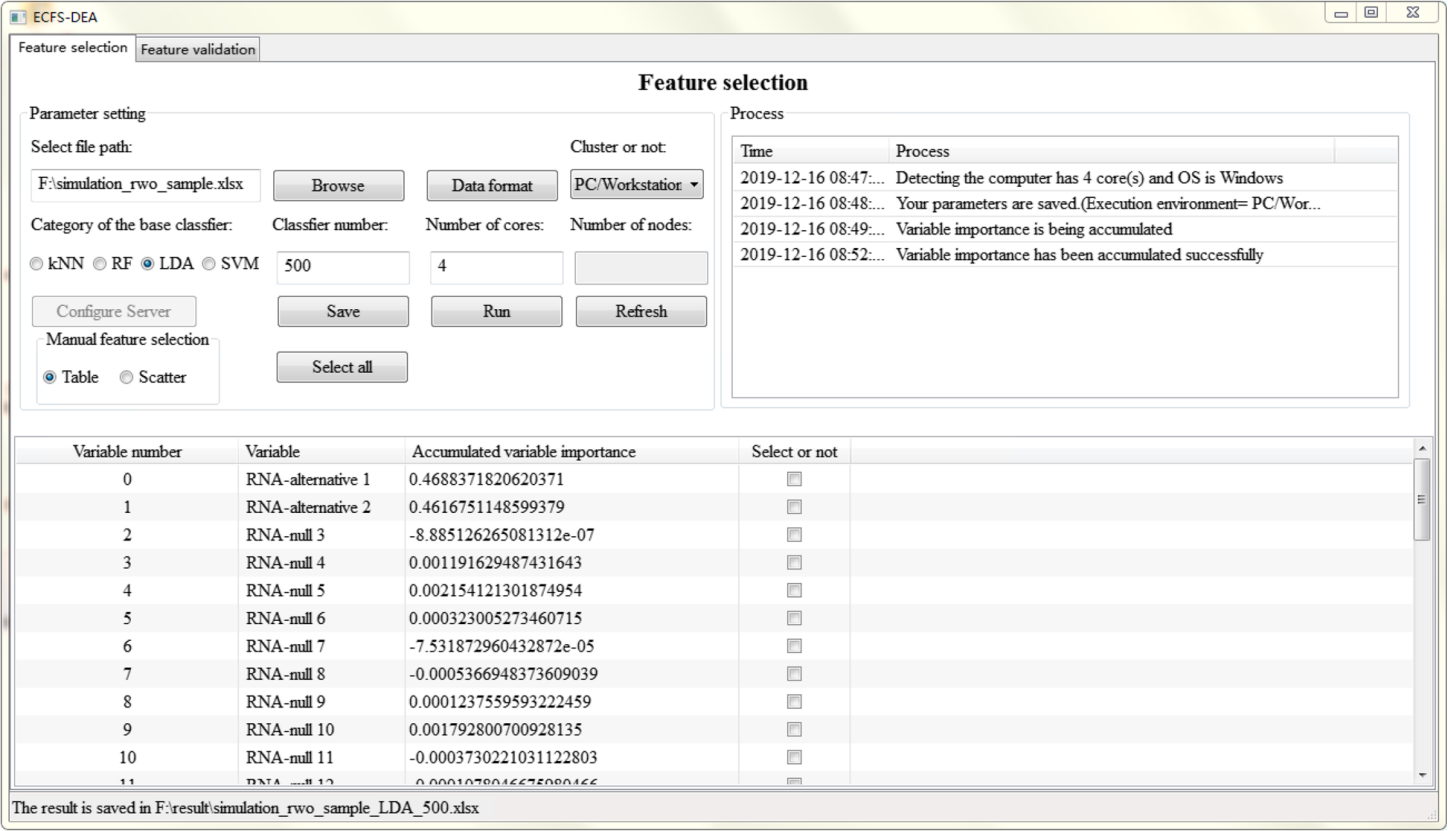
Feature selection step using a table form in ECFS-DEA

**Fig. 4:**
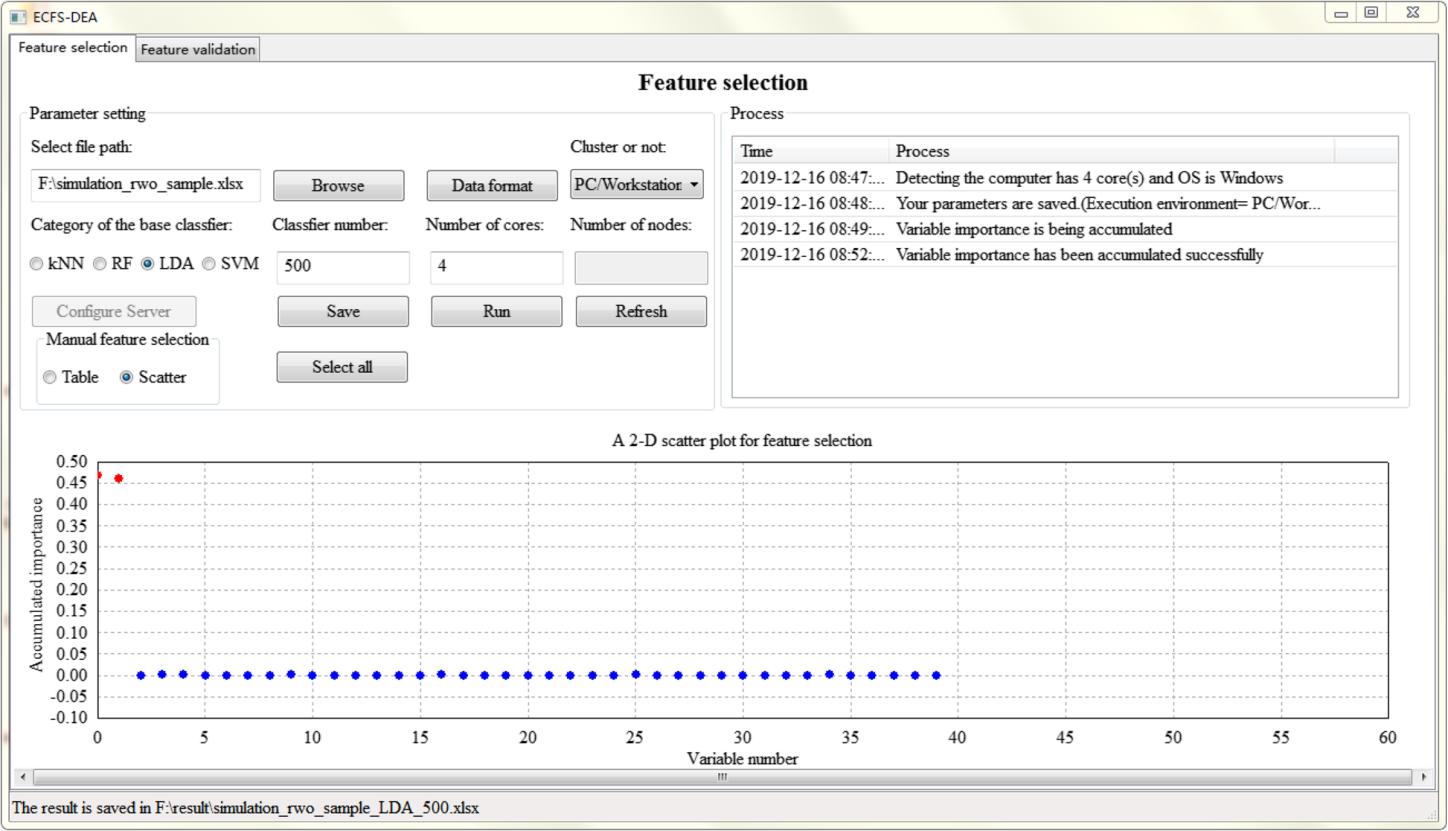
Feature selection step using a scatter plot in ECFS-DEA

The parameter setting step of feature selection part is illustrated in Fig. 2. The file path, data format, execution environment, etc. are set. Besides, the category of the base classifier is interactively assigned. The number of the base classifier which is also the resampling round needs to be appointed. Sample splitting is performed after parameter setting. Once the accumulation of variable importance is fulfilled, the obtained scores can be listed in a table or a scatter plot form for manual selection, as illustrated in Figs. 3 and 4 respectively.

In a table form as shown in Fig. 3, one can click the checkbox of the fourth column called “select or not” for fulfilling feature selection. The third column header can be clicked to rank. In a scatter plot form as shown in Fig. 4, one can double click the scatter to select the variable to be a part of a feature with its color changed red and vice versa. When users move the mouse around the scatter, the variable information can be displayed.

**Fig. 5:**
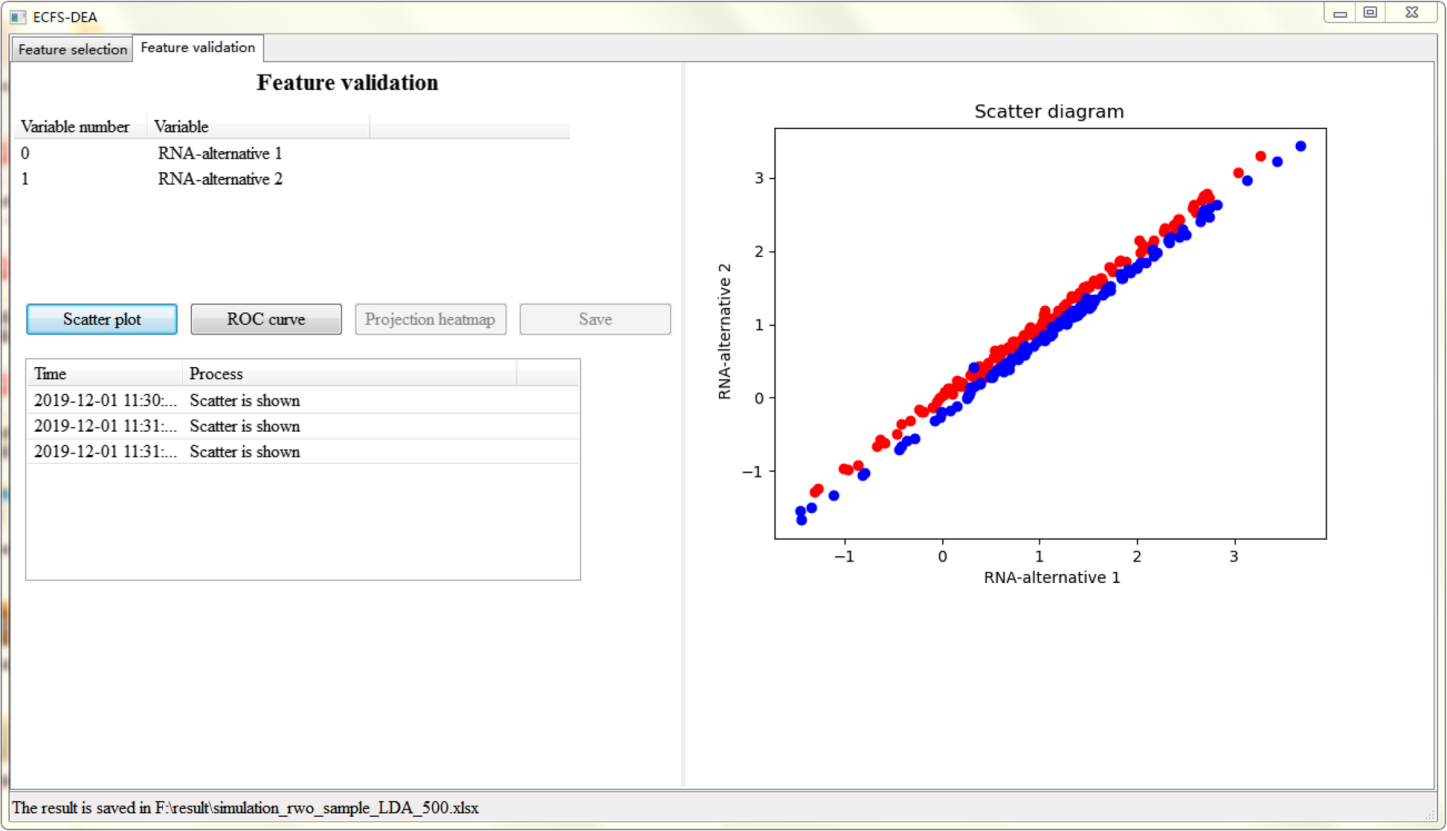
Feature validation step using a scatter plot in ECFS-DEA

**Fig. 6:**
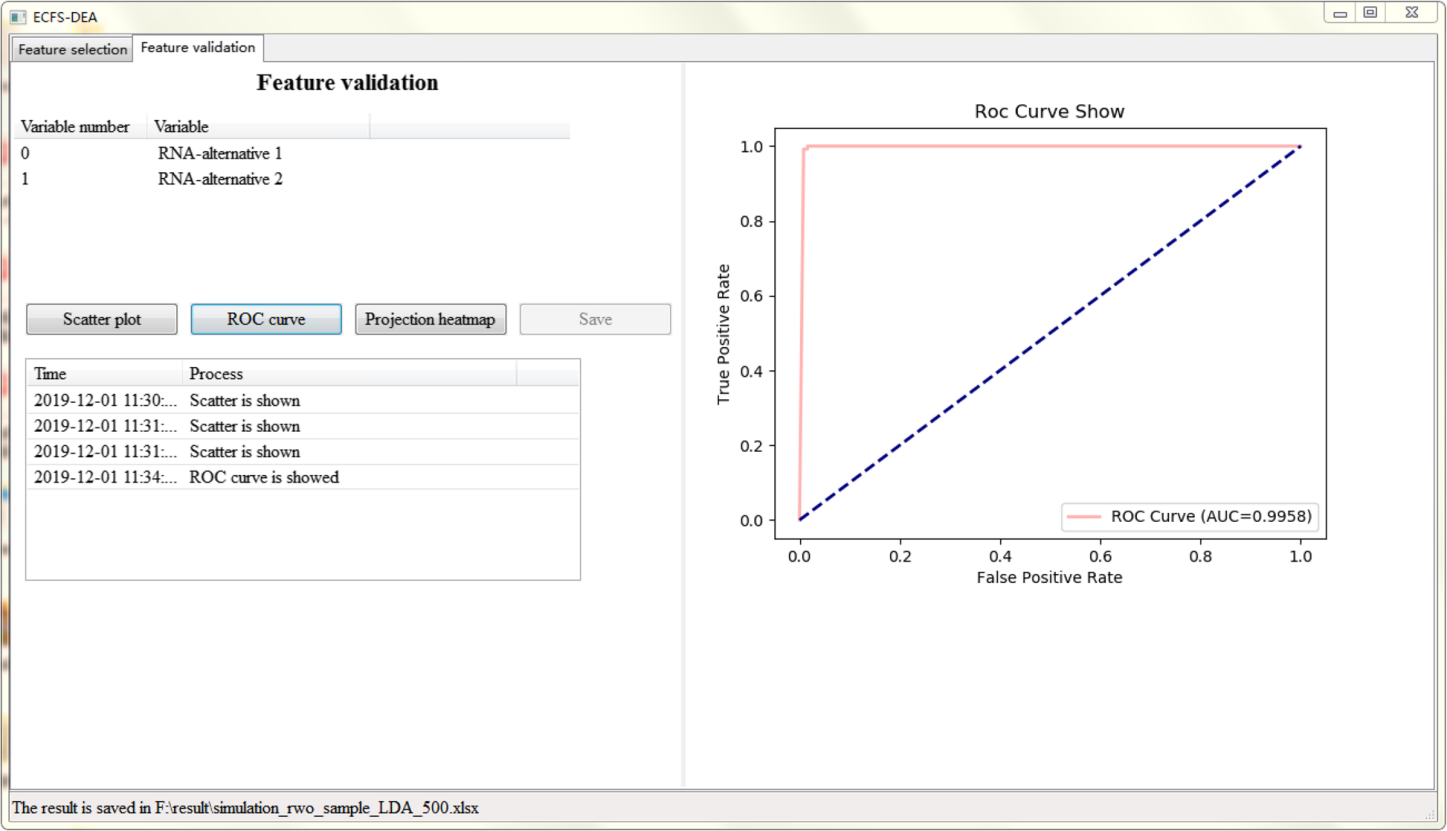
Feature validation step using a ROC curve in ECFS-DEA

**Fig. 7:**
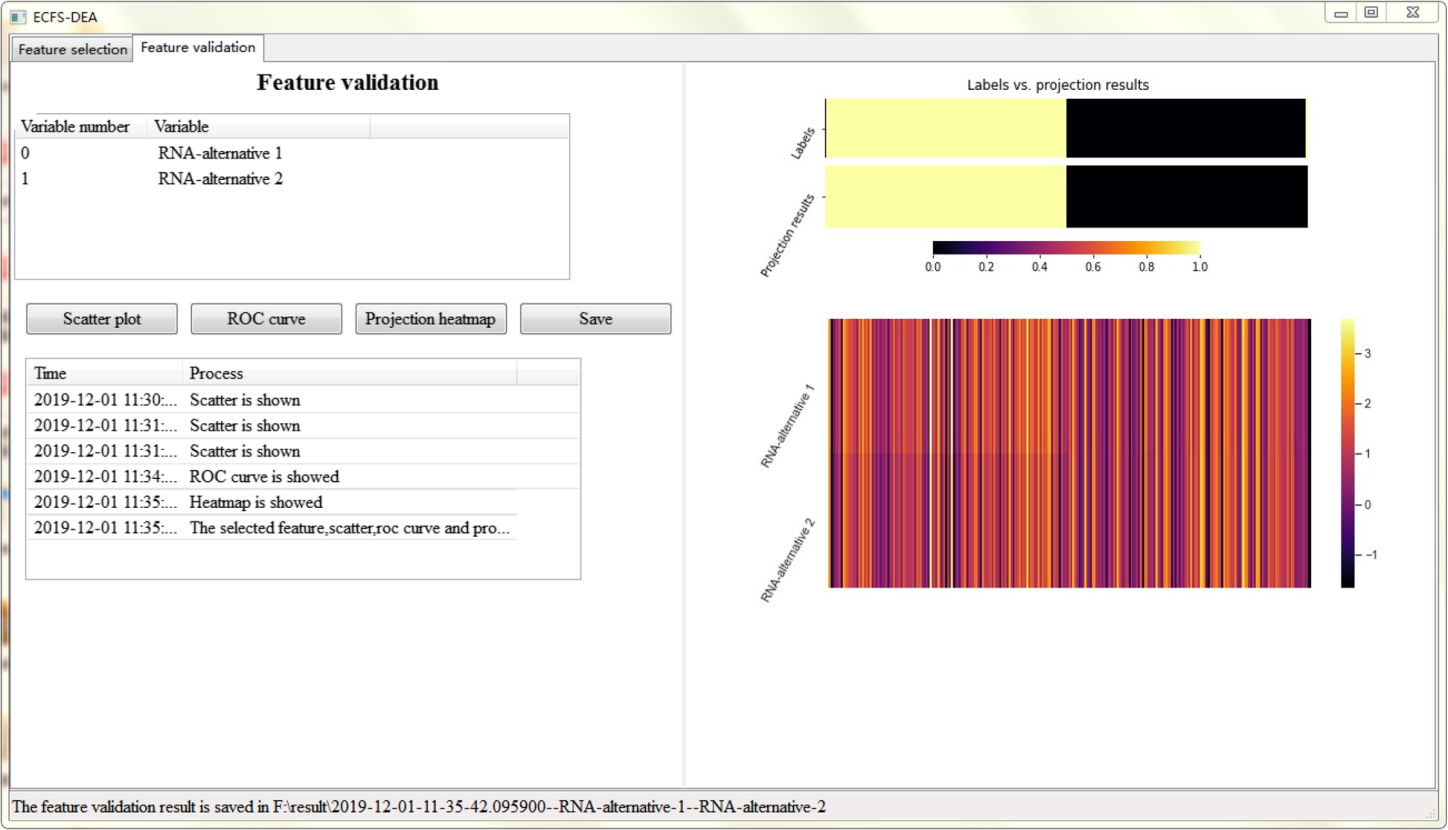
Feature validation step using a projection heatmap in ECFS-DEA

Figures 5, 6 and 7 together illustrate the panel for feature validation part of ECFS-DEA in Windows. Corresponding panels in Linux or Mac are almost the same. After pressing button “Scatter plot”, a 1-D, 2-D or 3-D scatter plot of the selected feature is shown in Fig. 5. Scatter plots with different colors denote samples from different groups. After pressing button “ROC curve”, the ROC curve of the selected feature is provided, as shown in Fig. 6. After pressing button “Projection heatmp”, the projection heatmap of the selected feature is presented, as shown in Fig. 7. A discrete projection from the expression levels of the selected feature (i.e., the classification results) is made. Samples are reordered according to the k-means clustering results of the projection values.

Detailed software documentation and tutorial are presented on http://bio-nefu.com/resource/ecfs-dea.

## Results

### Feature selection on the simulated data

In order to demonstrate the effectiveness of our ECFS-DEA, a simulated data consisting of 250 positive and 250 negative samples in a 40 dimensional space is constructed. 38 variables of them follow 38 normal distributions, each of which is independently and identically distributed and keeps a random mean value in range from 10 to 30 and a common standard deviation 0.01. The additional variable pair, i.e., *miRNA-alternative 1* and *miRNA-alternative 2*, follows a bivariate normal distribution and has a clear category distinction. The mean vectors corresponding to positive and negative samples are (1,1)^*T*^ and (1.11,0.89)^*T*^, respectively. Correspondingly, a same covariance matrix, which is expressed as $\left ({\begin {array}{*{20}{c}} 1&{0.999}\\ {0.999}&1 \end {array}} \right)$, is kept.

We made this simulated data in order to show the effectiveness of using LDA compared to RF. Considering the comparability with real data, we made the sample size to be 500. This data can be downloaded at http://bio-nefu.com/resource/ecfs-dea.

**Fig. 8:**
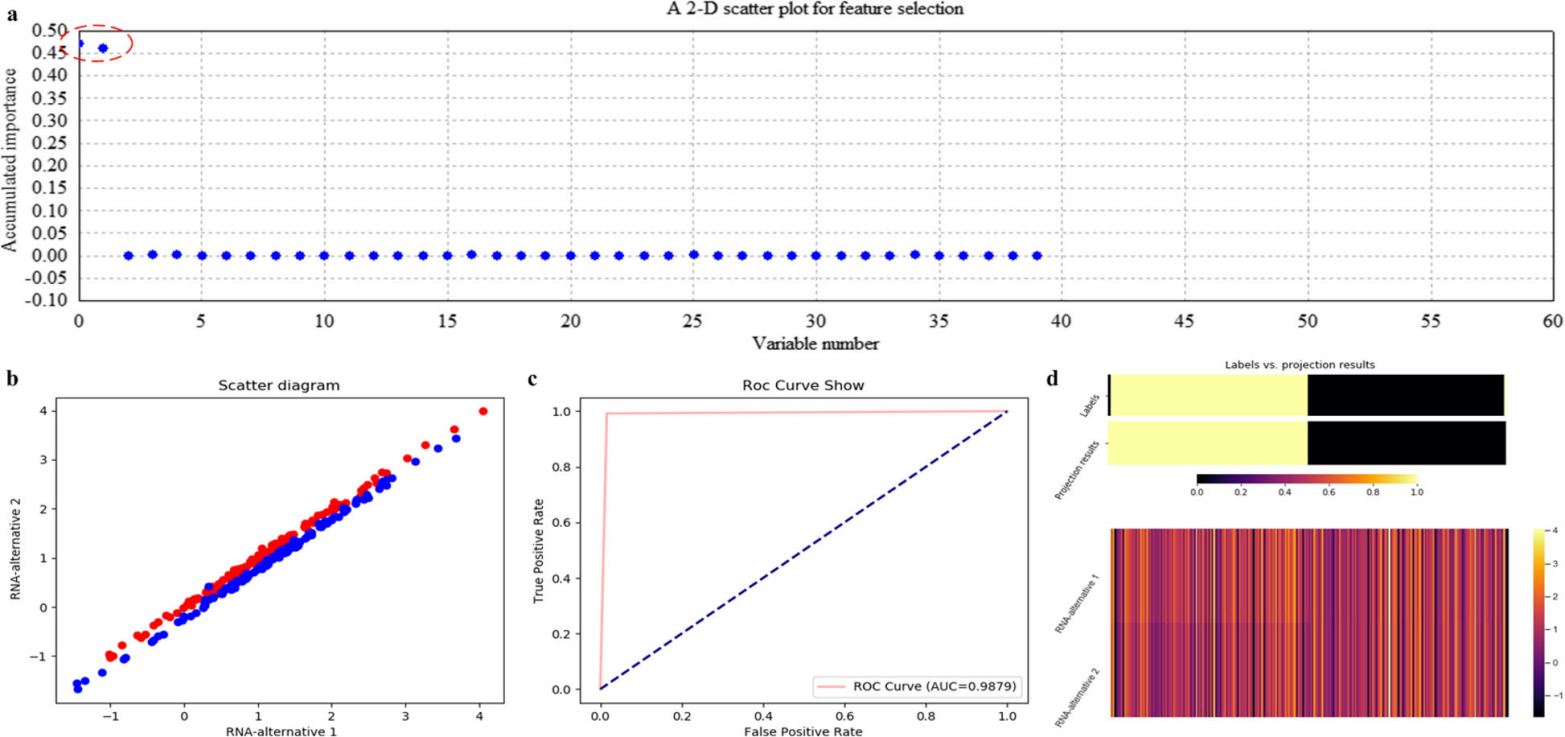
Feature selection and validation on the simulated data using LDA. **a** Feature selection in a scatter plot form. **b** The 2-D scatter plot. **c** The ROC curve. **d** The projection heatmap

Using ECFS-DEA with LDA assigned as the base classifier, the significant variable pair is properly selected on the training set according to the accumulation of variable importance after 500 rounds of resampling, as shown in Fig. 8a. Meanwhile, the corresponding 2-D scatter plot, the ROC curve and the projection heatmap of the testing group are illustrated in turn, as shown in Fig. 8b, c and d. It can be seen in Fig. 8b that the testing set is 2-D but not 1-D linearly separable. The corresponding ROC curve is shown in Fig. 8c. As to Fig. 8d, a discrete projection from the expression levels of the selected variable pair (i.e., the classification results) is made. Samples are reordered according to the k-means cluster results of the projection values. It can be seen in Fig. 8d that a sample labeled 0 is misclassified, which corresponds to the blue point within the points labeled red in Fig. 8b.

**Fig. 9:**
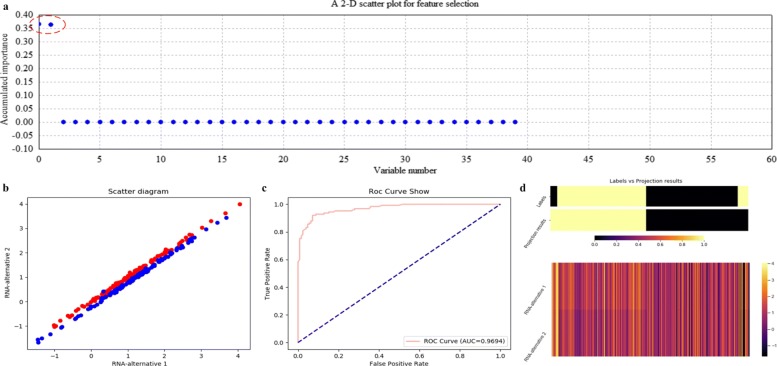
Feature selection and validation on the simulated data using kNN (k=5). **a** Feature selection in a scatter plot form. **b** The 2-D scatter plot. **c** The ROC curve. **d** The projection heatmap

Figure 9 illustrates the variable selection results using kNN (k =5) on the simulated data after 500 rounds of resampling. In Fig. 9a, miRNA-alternative 1 and miRNA-alternative 2 are also intuitively selected. Correspondingly, the scatter plot, the ROC curve and the projection heatmap are listed in Fig. 9b, c and d, which show the effectiveness of choosing kNN as the base classifier on the simulated data.

**Fig. 10:**
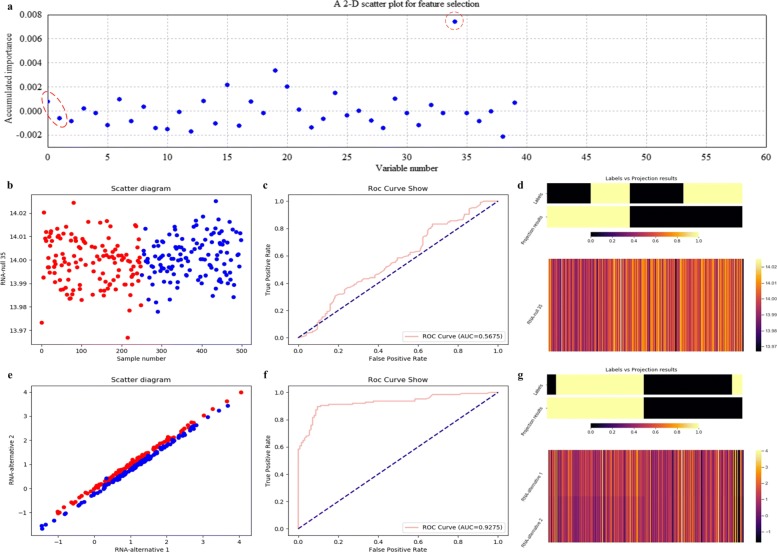
Feature selection and validation on the simulated data using RF. **a** Feature selection in a scatter plot form. **b** The 1-D scatter plot of the selected feature with x and y coordinates to be sample indices and expression values. **c** The ROC curve of the selected feature. **d** The projection heatmap of the selected feature. **e** The 2-D scatter plot of the significant pair. **f** The ROC curve of the significant pair. **g** The projection heatmap of the significant pair

Figure 10 illustrates the variable selection results using RF on the simulated data after 500 rounds of resampling. As shown Fig. 10a, it is miRNA-null 35 but not miRNA-alternative 1 and miRNA-alternative 2 that is selected. And it is considered as a false selection. This directly demonstrates that RF is not applicable to any data with different sample distributions. Correspondingly, the scatter plot, the ROC curve and the projection heatmap of miRNA-null 35 are listed in Fig. 10b, c and d. All these results further demonstrate the above phenomenon.

Figure 10b illustrates a 1-D scatter plot of the selected miRNA-null 35 using RF. The horizontal and vertical coordinates correspond to sample indices and expression levels, respectively. It can be seen that samples from two categories of the testing data are indivisible according to the vertical coordinate values. Figure 10c illustrates a poor ROC curve. As to Fig. 10d, it can be seen that the two clusters derived from the projection results contain many wrong labels.

Correspondingly, we also make the scatter plot, the ROC curve and the projection heatmap using RF on miRNA-alternative 1 and miRNA-alternative 2, which are listed in Fig. 10e, f and g, respectively. The experimental results of RF have improved; however, its ROC curve and projection heatmap are inferior to those of kNN and LDA.

**Fig. 11:**
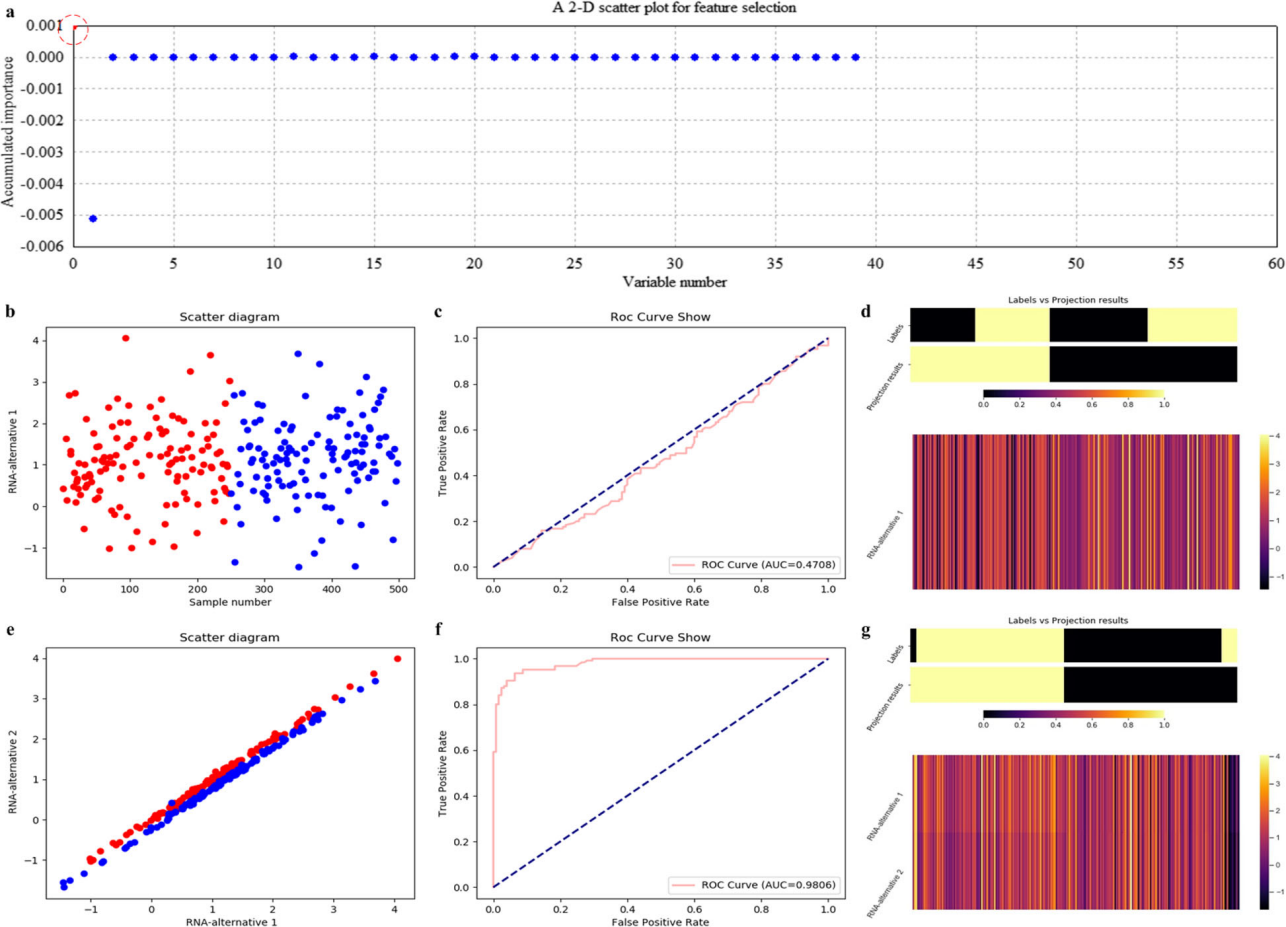
Feature selection and validation on the simulated data using SVM. **a** Feature selection in a scatter plot form. **b** The 1-D scatter plot of the selected feature with x and y coordinates to be sample indices and expression values. **c** The ROC curve of the selected feature. **d** The projection heatmap of the selected feature. **e** The 2-D scatter plot of the significant pair. **f** The ROC curve of the significant pair. **g** The projection heatmap of the significant pair

As to SVM which is assigned as the base classifier, it is only miRNA-alternative 1 but not the significant pair that is selected, as illustrated in Fig. 11a. It indicates that SVM is not applicable to the simulated data for feature selection. Correspondingly, the scatter plot, the ROC curve and the projection heatmap of miRNA-alternative 1 are listed in Fig. 11b, c and d. On the contrary, we also make the scatter plot, the ROC curve and the projection heatmap using SVM on miRNA-alternative 1 and miRNA-alternative 2, as shown in Fig. 11e, f and g.

The quantitative results on the simulated data with measures such as confusion matrix, precision, recall and F1-measure are listed in Table 1. In fact, it can be seen that RF and SVM achieve poor results, for they correspond to lower scores of accumulated importance compared with those of LDA and kNN, as shown in Figs. 8a, 9a, 10a and 11a, respectively. All the experimental results indicate that LDA is a more appropriate classifier for feature selection on the simulated data.

**Table 1:** Quantitative results on the simulation data

Base classifier	Variable number	Confusion matrix			Positive class	Precision	Recall	F1-measure
LDA	[0,1]^*T*^	classified as	a	b	a	0.992	0.984	0.988
		label a	123	2	b	0.984	0.992	0.988
		label b	1	124	weighted average	0.988	0.988	0.988
kNN	[0,1]^*T*^	classified as	a	b	a	0.906	0.928	0.917
		label a	116	9	b	0.926	0.904	0.915
		label b	12	113	weighted average	0.916	0.916	0.916
RF	34	classified as	a	b	a	0.528	0.448	0.485
		label a	56	69	b	0.521	0.600	0.558
		label b	50	75	weighted average	0.524	0.524	0.522
	[0,1]^*T*^	classified as	a	b	a	0.897	0.904	0.900
		label a	113	12	b	0.903	0.896	0.899
		label b	13	112	weighted average	0.900	0.900	0.899
SVM	0	classified as	a	b	a	0.467	0.400	0.431
		label a	50	75	b	0.476	0.544	0.508
		label b	57	68	weighted average	0.472	0.472	0.470
	[0,1]^*T*^	classified as	a	b	a	0.909	0.960	0.934
		label a	120	5	b	0.958	0.904	0.930
		label b	12	113	weighted average	0.933	0.932	0.932

### Feature selection on GSE22058

We also performed experiments on GSE22058 [29] which is a public dataset containing 96 samples associated with liver tumor and 96 samples corresponded to adjacent liver non-tumor. In order to achieve a predictive feature from the 220 miRNAs, we utilized ECFS-DEA on GSE22058, with the base classifier to be LDA, kNN, RF and SVM.

**Fig. 12:**
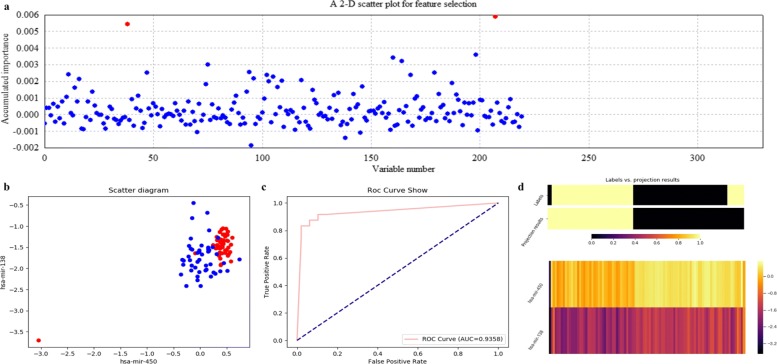
Feature selection and validation on GSE22058 using LDA. **a** Feature selection in a scatter plot form. **b** The 2-D scatter plot. **c** The ROC curve. **d** The projection heatmap

**Fig. 13:**
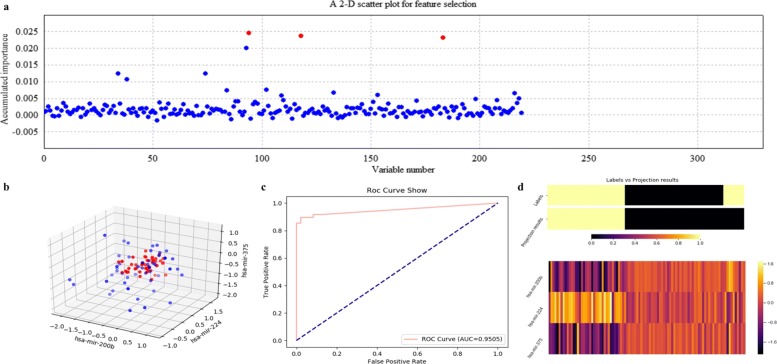
Feature selection and validation on GSE22058 using kNN (k=5). **a** Feature selection in a scatter plot form. **b** The 3-D scatter plot. **c** The ROC curve. **d** The projection heatmap

**Fig. 14:**
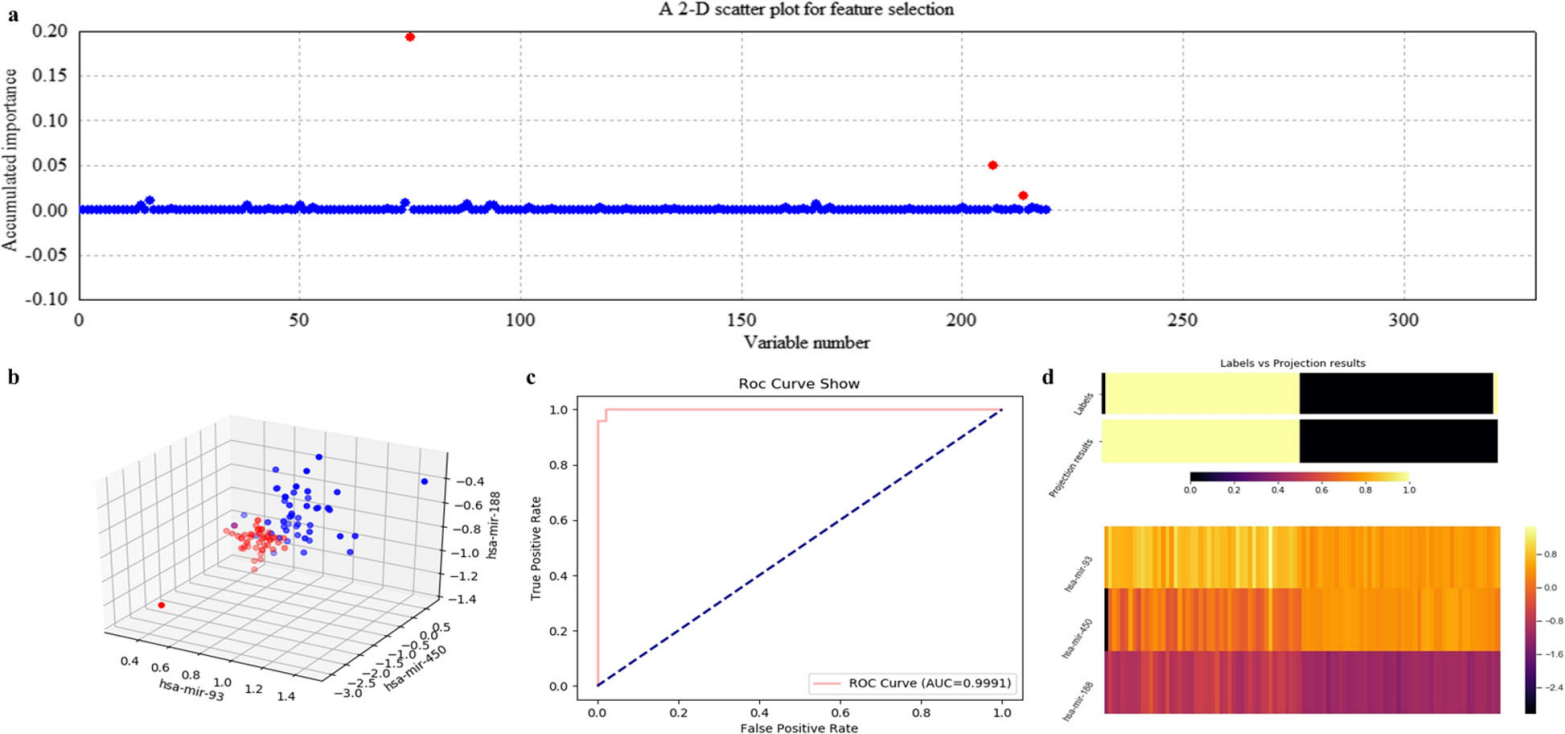
Feature selection and validation on GSE22058 using RF. **a** Feature selection in a scatter plot form. **b** The 3-D scatter plot. **c** The ROC curve. **d** The projection heatmap

**Fig. 15:**
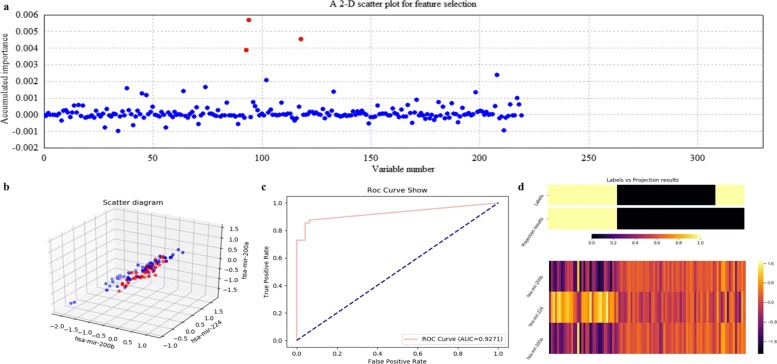
Feature selection and validation on GSE22058 using SVM. **a** Feature selection in a scatter plot form. **b** The 3-D scatter plot. **c** The ROC curve. **d** The projection heatmap

Figures 12, 13, 14 and 15 illustrate qualitative results for feature selection using LDA, kNN (k=5), RF and SVM on GSE22058 after 500 rounds of resampling, respectively. In order to exhibit the scatter plots at the feature validation step, we restricted feature dimension less than four. Besides, quantitative results on GSE22058 with measures such as confusion matrix, precision, recall and F1-measure are listed in Table 2, with all possible variables intuitively selected. All the experimental results indicate that RF is a more appropriate classifier to feature selection on GSE22058.

**Table 2:** Quantitative results on GSE22058

Base classifier	Variable number	Confusion matrix			Positive class	Precision	Recall	F1-measure
LDA	207	classified as	a	b	a	0.885	0.958	0.920
		label a	46	2	b	0.955	0.875	0.913
		label b	6	42	weighted average	0.920	0.916	0.917
	[207,38]^*T*^	classified as	a	b	a	0.852	0.958	0.902
		label a	46	2	b	0.952	0.833	0.889
		label b	8	40	weighted average	0.902	0.895	0.895
	[207,38,198]^*T*^	classified as	a	b	a	0.887	0.979	0.931
		label a	47	1	b	0.977	0.875	0.923
		label b	6	42	weighted average	0.932	0.927	0.927
	[207,38,198,160]^*T*^	classified as	a	b	a	0.922	0.979	0.950
		label a	47	1	b	0.978	0.917	0.947
		label b	4	44	weighted average	0.950	0.948	0.948
	[207,38,198,	classified as	a	b	a	0.922	0.979	0.950
		label a	47	1	b	0.978	0.917	0.947
	160,164]^*T*^	label b	4	44	weighted average	0.950	0.948	0.948
	[207,38,198,	classified as	a	b	a	0.904	0.979	0.940
		label a	47	1	b	0.977	0.896	0.935
	160,164,75]^*T*^	label b	5	43	weighted average	0.941	0.938	0.938
kNN	94	classified as	a	b	a	0.730	0.958	0.829
		label a	46	2	b	0.939	0.646	0.765
		label b	17	31	weighted average	0.835	0.802	0.797
	[94,118]^*T*^	classified as	a	b	a	0.800	1.000	0.889
		label a	48	0	b	1.000	0.750	0.857
		label b	12	36	weighted average	0.900	0.875	0.873
	[94,118,183]^*T*^	classified as	a	b	a	0.828	1.000	0.906
		label a	48	0	b	1.000	0.792	0.884
		label b	10	38	weighted average	0.914	0.896	0.895
	[94,118,183,93]^*T*^	classified as	a	b	a	0.787	1.000	0.881
		label a	48	0	b	1.000	0.729	0.843
		label b	13	35	weighted average	0.893	0.865	0.862
RF	75	classified as	a	b	a	0.904	0.979	0.940
		label a	47	1	b	0.977	0.896	0.935
		label b	5	43	weighted average	0.941	0.938	0.938
	[75,207]^*T*^	classified as	a	b	a	0.979	0.979	0.979
		label a	47	1	b	0.979	0.979	0.979
		label b	1	47	weighted average	0.979	0.979	0.979
	[75,207,214]^*T*^	classified as	a	b	a	0.979	0.979	0.979
		label a	47	1	b	0.979	0.979	0.979
		label b	1	47	weighted average	0.979	0.979	0.979
	[75,207,214,16]^*T*^	classified as	a	b	a	0.980	1.000	0.990
		label a	48	0	b	1.000	0.979	0.989
		label b	1	47	weighted average	0.990	0.990	0.990
SVM	94	classified as	a	b	a	0.746	0.979	0.847
		label a	47	1	b	0.970	0.667	0.790
		label b	16	32	weighted average	0.858	0.823	0.819
	[94,118]^*T*^	classified as	a	b	a	0.787	1.000	0.881
		label a	48	0	b	1.000	0.729	0.843
		label b	13	35	weighted average	0.893	0.865	0.862
	[94,118,93]^*T*^	classified as	a	b	a	0.774	1.000	0.873
		label a	48	0	b	1.000	0.708	0.829
		label b	14	34	weighted average	0.887	0.854	0.851

In addition, we searched the selected miRNAs using ECFS-DEA with RF to be the classifier, i.e., miR-188, miR-450 and miR-93, on Web of Science with keywords to be such as *liver tumor*, *hepatocellular carcinoma* and *HCC*. Both miR-188 and miR-93 have been reported to be relevant to liver tumor. In fact, miR-188 achieved higher scores than other miRNAs, as shown in Fig. 14a. The retrieved results of miR-188 [30, 31] have indirectly demonstrated the effectiveness of ECFS-DEA.

## Conclusions

ECFS-DEA is a top-down classification-based tool for seeking predictive variables associated with different categories of samples on expression profiles. Other than prevailing differential expression analysis for class prediction, an ensemble classifier-based thought is proposed in this paper. According to accumulated scores of variable importance, LDA, kNN, RF or SVM can be rightly assigned and is suitable for different sample distributions. Qualitative and quantitative experimental results have demonstrated the effectiveness of ECFS-DEA.

## Availability and requirements

**Project name**: ECFS-DEA **Project home page**: http://bio-nefu.com/resource/ecfs-dea**Operating system(s)**: Linux, Windows, Mac **Programming language**: Python (≥ 3.5) **License**: GPLv3 **Any restrictions to use by non-academics**: none

## Data Availability

The public dataset analysed during the current study is available in the GEO repository. GSE22058 is available at https://www.ncbi.nlm.nih.gov/geo/query/acc.cgi?acc=GSE22058. The simulated data can be downloaded on http://bio-nefu.com/resource/ecfs-dea.

## Abbreviations

DEA: Differential expression analysis
ECFS-DEA: Ensemble classifier-based feature selection for differential expression analysis
JCD-DEA: Joint covariate detection for differential expression analysis
kNN: k-nearest-neighbor
LDA: Fisher’s linear discriminative analysis
PBS: Portable batch system
RF: Random forest
ROC: Receiver operating characteristic
SAM: Significance analysis of microarrays
